# The Effect of C-X-C Motif Chemokine 13 on Hepatocellular Carcinoma Associates with Wnt Signaling

**DOI:** 10.1155/2015/345413

**Published:** 2015-06-16

**Authors:** Chunyan Li, Dong Kang, Xiguang Sun, Yufei Liu, Jinhong Wang, Pujun Gao

**Affiliations:** ^1^Department of Pediatric Respiratory, The First Affiliated Hospital of Jilin University, Changchun 130000, China; ^2^Huiqiao Department, South Hospital of Southern Medical University, Guangdong 510000, China; ^3^Department of Hand Surgery, The First Affiliated Hospital of Jilin University, Changchun 130000, China; ^4^Pediatric Digestive Department, The First Affiliated Hospital of Jilin University, Changchun 130000, China; ^5^Department of Respiratory, The Third Affiliated Hospital of Southern Medical University, Guangdong 510000, China; ^6^Division of Hepatobiliary and Pancreatic Diseases, The First Affiliated Hospital of Jilin University, Changchun 130000, China

## Abstract

*Objects*. To investigate the effect of CXCL13 (C-X-C motif chemokine 13) on hepatocellular carcinoma and clarify the potential mechanisms. *Methods*. 32 patients with hepatocellular carcinoma and 12 healthy controls were recruited for analyzing the expression of CXCL13 by RT-PCR (reverse transcription-polymerase chain reaction). ELISA (enzyme-linked immune-sorbent assay) was used to test the concentration of serum CXCL13. The interaction between CXCL13 and Wnt signaling was analyzed by western blot. In vitro PBMCs cultured with HepG2 supernatant, the levels of IL-12, IL4, IL-6, and IL-17, and four IgG subclasses were detected by ELISA. *Results*. The rate of high expression CXCL13 was 63.4% in advanced HCC patients, and the serum CXCL13 was also at a high level in stage IV HCC patients. Meanwhile CXCL13 level was positively correlated with serum ALT (Alanine Transaminase) and AST (Aspartate Aminotransferase). CXCL13 and Wnt/*β*-catenin signaling shared a positive feedback loop. Furthermore, CXCL13 could obviously promote the expressions of IL-12 and IL-17, and induce IgG4 secreted by B cells. *Conclusions*. The effect of CXCL13 on promoting liver cancer is related to the activation of Wnt/*β*-catenin pathway and the facilitation of IL-12, IL-17 and IgG4. CXCL13 plays an important role in the progression of HCC, and it may act as a potential target for the diagnosis and treatment of HCC.

## 1. Introduction

Hepatocellular carcinoma (HCC) is the most primary malignant tumor of the liver cells (with more than 750,000 new cases diagnosed every year worldwide) and the third most deadly tumor globally, following lung and stomach cancers [1]. Unlike other carcinomas, the mortality from most malignancies has decreased steadily in the last 20 years, however, that from liver cancer has increased significantly from 1990 to 2005, by as much as 50% in men [2]. Though the main risk factors for HCC development have been clearly identified, such as hepatitis B and C virus infection, alcohol abuse, and some chronic liver diseases [3, 4], there is still preliminary understanding of the key drivers of this malignancy. Another lethal problem of HCC is the symptoms of early-stage HCC which are often not apparent, so many patients are diagnosed at advanced stages [5, 6], leading to less effective therapies. What is more, though antivirals and vaccination could effectively decrease the incidence of HCC, there is not widely accepted chemopreventive strategy to limit development of HCC once cirrhosis is established [7, 8]. Limited treatment options highlight the need to clarify the mechanisms in HCC development and to identify early disease biomarkers and new therapy targets.

The molecular pathogenesis of HCC is relative to multiple influences such as tumor microenvironment [9, 10] and abnormal activation of some signaling pathways [11, 12]. Several clear evidences have confirmed that chemokine/chemokine receptor in the inflammatory interactions plays critical roles in tumorigenesis and metastasis. The chemokines are a group of small (<15 kDa), soluble proteins that bind to their G-protein-coupled receptors to mediate different pro- and anti-inflammatory responses [13–15]. Based on the position of the key cysteine residues located in the N-terminal region, chemokines are subdivided into four families: CXC, CC, C, and CX3C chemokines, in which the X represents any amino acid [16]. Chemokines play an essential role in tumor progression, acting on endothelial, epithelial, and tumor cells. They are reported to sustain tumor cell growth, induce angiogenesis, and facilitate tumor escape through evasion of immune surveillance [17–19]. Several chemokines/chemokines receptors appear to be directly implicated in HCC. The CCL20-CCR6 axis may mediate the growth and progression of HCC through phosphorylation of MAPK [20, 21] and is associated with poor prognosis after resection [22, 23]. CCL5-CCR1 promotes metastasis and invasion of the HCC cell line Huh7 and CCL3-CCR1 contributes to the growth and progression of HCC, whereas CX3CL1-CX3CR1 axis is believed to be involved in HCC tumor growth inhibition [24]. CXCL12-CXCR4 axis plays a critical role in migration of tumor cells into metastatic sites in HCC [25].

CXCL13 (B-lymphocyte chemoattractant (BLC)), which is the only chemokine binding to its receptor CXCR5, is mainly secreted by follicular dendritic cells (FDCs), monocyte-like mature macrophages, and stromal cells in the B-cell area of the secondary lymphoid tissues [26, 27], where the B cells encounter the antigen and differentiate [28]. CXCL13 may contribute significantly to breast tumor formation and mediate the progress of prostate cancer through activating JNK and ERK pathways [29–31]. However, the effect of CXCL13 in HCC has not yet been clarified clearly.

In this study, we confirmed the high level CXCL13 was existed in both tissue and serum of advanced liver cancer patients. We also found the activation of Wnt/*β*-catenin signaling could increase the expressions of CXCL13 and its receptor CXCR5, while CXCL13 could stimulate the Wnt/*β*-catenin pathway. By culturing PBMCs with HepG2 culture supernatant, CXCL13 was identified to significantly induce the production of IL-12 and IL-17 secreted by T cells, and moreover, it could upregulate the concentration of IgG4 secreted by B cells. These results indicated that the effect of CXCL13 on promoting liver cancer development might be related to the activation of Wnt/*β*-catenin pathway and the facilitation of IL-12, IL-17 and IgG4. CXCL13 played an important role in the progression of HCC, and it might serve as a potential target for the diagnosis and treatment of HCC.

## 2. Materials and Methods

### 2.1. Patients

A total of 32 patients with new onset HCC and 12 gender- and age-matched health controls (HC) were recruited from the Hepatopancreatobiliary Department of the First Hospital of Jilin University (Changchun, China). To avoid the interference of other diseases, the participators with any autoimmune diseases, hepatitis virus infection, or those who had received drug therapies within the past 6 months were excluded from the study. Written informed consent was obtained from individual subjects and the experimental protocol was approved by the Ethical Committee of the First Hospital of Jilin University. Their demographic and clinical characteristics of these subjects are show in Table 1. The levels of serum AST (Aspartate Aminotransferase), ALT (Alanine Transaminase), and AFP (Alpha Fetal Protein) in participants were detected by Biochemistry Automatic Analyzer (Roche Diagnostics, Branchburg, USA).

Fresh liver tumor samples were collected immediately after surgical resection and stored in liquid nitrogen until further use. The corresponding serum samples were stored in −80°C freezer.

### 2.2. Isolation of PBMCs and Cell Culture In Vitro

Peripheral blood mononuclear cells (PBMCs) from venous blood samples (10 mL) of health controls were sorted by Ficoll-Paque (Amersham Biosciences, USA) density-gradient centrifugation. PBMCs were washed three times in Hanks balanced salt solution (Gibco, Canada), counted, and suspended in Dulbecco's Modified Eagle Medium (DMEM, Invitrogen), containing 10% FBS (Gibco, USA) and penicillin streptomycin solution (Hyclone, USA). The liver cancer cell line HepG2 was obtained from the Cell Bank of the Chinese Academy of Sciences (Shanghai, China) and cultured as mentioned above.

To analyze the effect of CXCL13 on the immune status of HCC, PBMCs cells (5 × 10^5^ cells/well) were seeded in a 24-well plate (Corning, USA), culturing using the supernatant of HepG2 cells. After 72 h, the supernatant was taken and levels of cytokines and IgG subclasses were determined by ELISA. For the proliferation of T lymphocytes, PBMCs were initially cultured in a 24-well plate precoated with CD3/CD28 mAbs (1 *μ*g/mL CD3 and 10 *μ*g/mL CD28 resp., both from invitrogen, USA). To activate B cells, 3 *μ*g/mL CpGB and 10 ng/mL IL-2 (both from R&D Systems, USA) were used to stimulate B cells for 72 h.

100 ng/mL DKK-1 (ACROBiosystems, USA) was obtained from PeproTech, using to generally block Wnt/*β*-catenin pathways, and 100 mM LiCl (Nacarai Tesque, Japan) acted as a stimulator of this pathway. Recombinant human CXCL13 (100 ng/mL) [31, 32] was from R&D, USA. Dexamethasone (0.2 mg/mL) was used to inhibit CXCL13 expression [33].

### 2.3. Enzyme-Linked Immune-Sorbent Assay (ELISA)

According to the manufacturer's instructions, enzyme-linked immune-sorbent assay (ELISA) of serum and cell culture supernatant was performed on 96-well plates. The serum or supernatant levels of human CXCL13 and IL-12/4/6/17 were determined by ELISA kit from R&D, USA. The concentrations of IgG1, IgG2, IgG3, IgG4, and total IgG were determined by ELISA (Uscn Life Science, China) as described previously [34, 35]. The absorbance of the plates was read at 450 nm using an Automated Microplated Reader (Bio-Tek, USA).

### 2.4. Analysis of mRNA Levels by Reverse Transcription (RT) PCR

To test the mRNA expression in liver cancer tissues, total RNA from HCC tissues were extracted using TRIzol (Invitrogen, USA) according to the manufacturer's instructions. RNA pellets were stored in sterile ribonuclease-free water. Reverse transcription was carried out using 1–3 *μ*g total RNA, 0.5 *μ*g oligo (dT), and Superscript II enzyme (Invitrogen, USA). The gene-specific primers for RT-PCR were listed as follows: CXCL13 forward 5′-CTGGTCAGCAGCCTCTCTC-3′, and reverse 5′-TTCTCAATACTTCCATCATTCTTT-3′; GAPDH forward 5′-CACCAACTGGGACGACAT-3′, and reverse 5′-ACAGCCTGGATAGCAACG-3′. The mRNA expression of interested gene in each sample was normalized against housekeeping gene.

### 2.5. Western Blotting

To illustrate the interaction between CXCL13 and Wnt pathway, HepG2 cells were plated in 6-well plates (3 × 10^6^ cells/well). After 8 h treatment, cells were harvested and lysed on ice for 30 min in RIPA (50 mM Tris-HCl pH 7.4, 150 mM NaCl, 0.1% SDS, 1% deoxycholate, 1% TritonX-100, 1 mM EDTA, 5 mM NaF, 1 mM sodium vanadate, and protease inhibitors cocktail) buffer and protein extracts were quantitated. 20 *μ*g of total protein was then subjected to 10–15% SDS-PAGE, electrophoresed and transferred to a nitrocellulose membrane. After using 5% nonfat milk in Tris-buffered saline for blocking, the membrane was washed and incubated with the indicated antibodies. The primary antibodies for *β*-catenin, phospho-GSK-3*β* (Ser9), GSK3*β*, CXCL13, CXCR5, and GAPDH were all from Santa Cruz, USA. The animal-matched horseradish peroxidase-conjugated secondary antibody was purchased from Santa Cruz as well.

### 2.6. Statistical Analysis

Data are expressed as median and range or individual mean values. The difference between the groups was analyzed by Mann-Whitney test using the Graphpad 5.0 software. The relationship between variables was evaluated using the Spearman rank correlation test. A two-side *P* value of <0.05 was considered statistically significant.

## 3. Results

### 3.1. Serum Level of CXCL13 Was Increased in Liver Cancer Tissues and Was Relative to the Development of HCC

**Figure 1:**
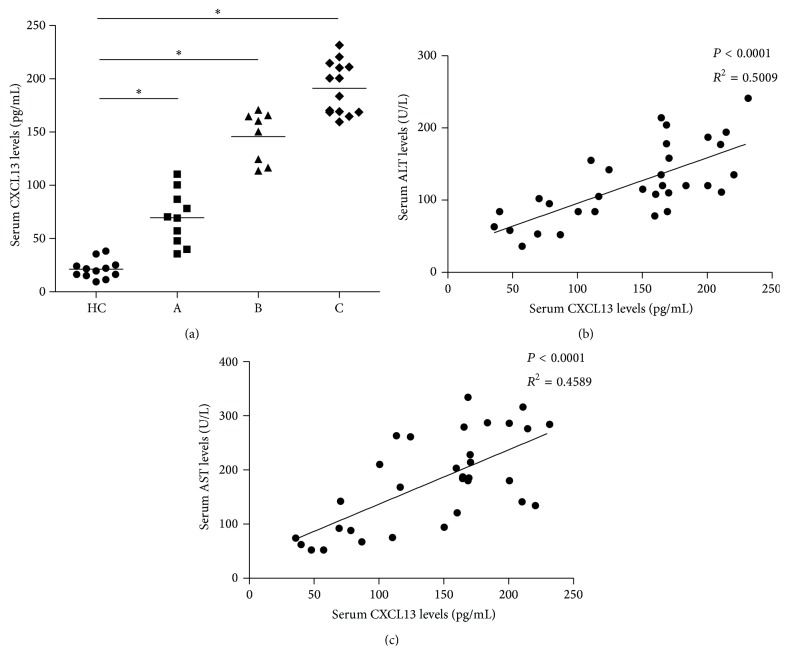
The concentration of serum CXCL13 in liver cancer patients. (a) The serum CXCL13 level in 32 HCC patients and 12 health controls detected by ELISA. CXCL13 level was much higher in stage IV HCC patients than that in other groups. There also existed statistical significance between serum CXCL13 in stage III and stages I-II group (^*∗*^ *P* < 0.05). The horizontal lines indicate the median values for each group. (b-c) The potential correlations between CXCL13 and serum ALT and AST were analyzed by the Spearman correlation tests.

As a potent chemokine mainly secreted by monocytes, lymphocytes and dendritic cells, CXCL13 could also be expressed in liver tissues as we tested. The mRNA level of CXCL13 was obviously higher in most of the stage IV liver cancer patients (63.4%) than that in health control as shown in Table 1. To confirm the serum level of CXCL13, we detected its concentration in stage I-II group: A, stage III group: B, stage IV group: C, and health controls: HC. The ELISA results indicated that serum CXCL13 was significantly higher in all HCC groups compared with HC. In particular, the concentration of CXCL13 in group C was much higher than that in group B, and likewise, CXCL13 in group B was higher than that in group A as shown in Figure 1(a). Furthermore, we also analyzed the relationship between CXCL13 and clinical features of liver cancer patients. A positive correlation was found between the concentrations of serum CXCL13 and ALT (*P* < 0.0001, *r* ^2^ = 0.5009) or AST (*P* < 0.0001, *r* ^2^ = 0.4589). No other obvious difference was observed in any of the clinical parameters (Figures 1(b) and 1(c)). Thus the serum CXCL13 level was associated with the progression of HCC, and it might be a potential biomarker for liver cancer progression.

### 3.2. The Mutual Promotion between CXCL13 and Wnt/*β*-Catenin Signaling

**Figure 2:**
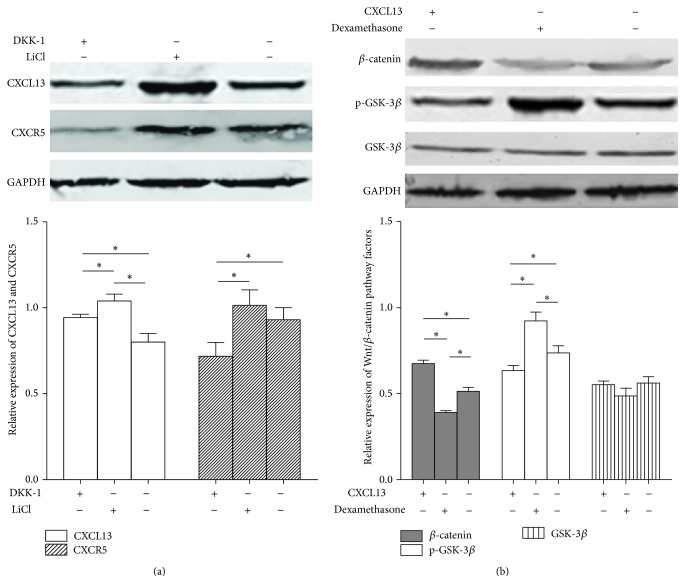
The interaction between CXCL13 and Wnt/*β*-catenin pathway. (a) The effect of Wnt/*β*-catenin signaling on CXCL13 and its receptor CXCR5 was analyzed by adding DKK-1 or LiCl to inhibit or stimulate the pathway, respectively. (b) The effect of CXCL13 on Wnt/*β*-catenin signaling. Dexamethasone was used to inhibit CXCL13. Gene expression levels were determined with GAPDH (glyceraldehyde 3-phosphate dehydrogenase) as the reference. A positive feedback loop was observed between CXCL13 and Wnt/*β*-catenin signaling (^*∗*^ *P* < 0.05). Experiments were performed in triplicate and repeated three times.

Though the regulatory role of CXCL13 has been proven to mediate the activation of JNK and MAPK pathways [30, 31] in prostate cancer invasion and migration, its modulating acts on Wnt/*β*-catenin signaling in HCC and the effect of Wnt/*β*-catenin pathway on CXCL13 was still obscure. In HepG2 cells, by treated with Wnt/*β*-catenin inhibitor DKK-1, we found decreased expressions of CXCL13 and CXCR5 compared with that in control, whereas upregulated levels of CXCL13 and CXCR5 were observed in stimulant LiCl treated group as shown in Figure 2(a). Furthermore, to investigate the influence of CXCL13 on this signaling, dexamethasone was added into the HepG2 cell medium to inhibit CXCL13. As we predicted, a decreased *β*-catenin and an increased p-GSK-3*β* expression were observed in the dexamethasone treated group, while inverse expressions of these two factors were founded in the CXCL13 stimulated group (Figure 2(b)). The results demonstrated there existed a positive relationship between CXCL13 and Wnt/*β*-catenin signaling in HCC, and this mutual interaction might promote the level of each other and led to the tumor microenvironment formation.

### 3.3. CXCL13 Upregulated the Concentrations of IL-12 and IL-17 in HCC

**Figure 3:**
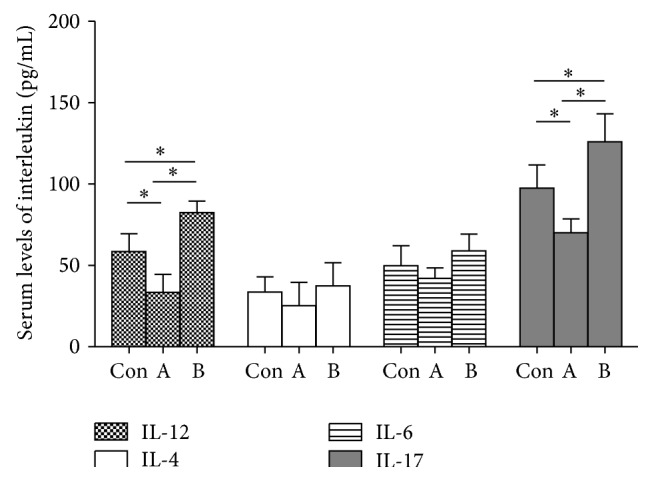
The influence of CXCL13 on T lymphocytes related inflammatory cytokines. PBMCs (5 × 10^5^/well) were isolated from health volunteers and seeded in 24-well plate precoated with CD3/CD28 mAbs. By culturing with HepG2 supernatant for 72 h, the concentrations of IL-12, IL-4, IL-6, and IL-17 were detected by ELISA. Meanwhile, PBMCs cultured with no stimulus were set to be negative control. The levels of IL-12 and IL-17 were obviously promoted by CXCL13 (^*∗*^ *P* < 0.05). Experiments were performed in triplicate and repeated three times.

CXCL13 has been revealed to play important roles in the immune response such as regulating lymphocyte migration and promoting inflammation; thus we further detected the influence of CXCL13 on immune status relative interleukins—IL-12, IL-4, IL-6, and IL-17 in three groups: dexamethasone group: A, CXCL13 group: B, and PBMCs control group. Cytokines in PBMCs which were cultured with HepG2 supernatant were collected and measured by ELISA. The concentrations of IL-12 and IL-17 in supernatant were significantly increased in CXCL13 stimulated group, while decreased in dexamethasone treated group (*P* < 0.05, Figure 3). But the levels of IL-4 and IL-6 were not statistically significant. Thus we suspected the proinflammatory effect of CXCL13 which was potentially mediated by triggering the expressions of cytokines, IL-12 and IL-17, which presented for the activation of Th1 and Th17 cells, respectively.

### 3.4. CXCL13 Significantly Improved IgG4 Secreting by B Cells

**Figure 4:**
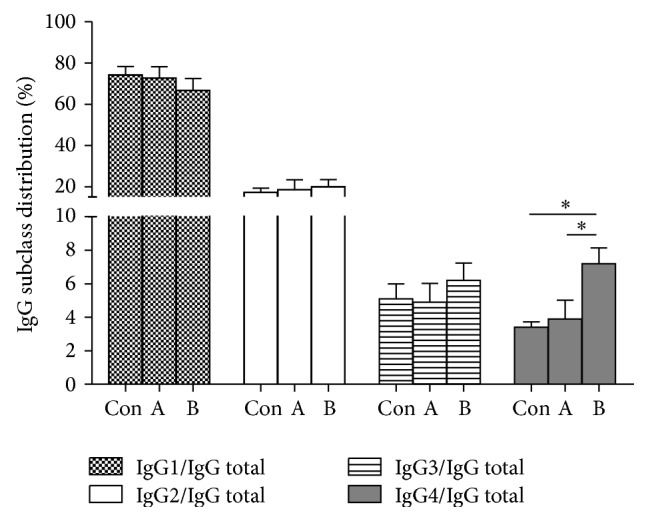
The effect of CXCL13 on B cells polarization. PBMCs (5 × 10^5^/well) were cultured in 24-well plate and stimulated using CpGB and IL-2 for 72 h to obtain activated B cells. The concentrations of IgG1, IgG2, IgG3, IgG4, and IgG total were detected using ELISA kits. PBMCs cultured with no stimulus were set to be negative control. The ratio of IgG4/IgG was obviously higher than that in other groups, while the ratios of IgG1/IgG, IgG2/IgG, and IgG3/IgG showed no statistical significances (^*∗*^ *P* < 0.05). Experiments were performed in triplicate and repeated three times.

Functionally, CXCL13 was initially acted as a selective chemoattractant for B lymphocytes and B helper T cells via its specific receptor CXCR5 and secreted by the stromal cells in the B-cell area of the secondary lymphoid tissues, where the B cells encounter the antigen and differentiate. Considering the influence of CXCL13 on promoting B-cell maturation, we next examined the proportions of IgG subclasses in PBMCs cultured with HepG2 supernatant. The groups divided as follows: dexamethasone group: A, CXCL13 group: B, and PBMCs with no additives which were set to be control group. After CpGB and IL-2 stimulating, we detected significantly increased IgG4/IgG total ratios in CXCL13 group as shown in Figure 4. These results indicated that CXCL13 could improve the polarized expression of IgG4 secreted by B cells. The high level of IgG4+ B cells may participate in the invasion and metastasis of liver cancer.

## 4. Discussion

The pathogenesis of HCC has been extensively analyzed to be closely associated with chronic inflammation which is mediated by inflammatory cytokines and chemotactic factors. In this study, our observation of elevated rate of high level CXCL13 in advanced liver cancer patients indicated a very close relation between CXCL13 and liver cancer. Further we proved that the concentration of serum CXCL13 was associated with HCC progression and positively correlated with serum ALT and AST. These findings implied the potential role of CXCL13 to be a biomarker for the pathogenesis of HCC.

The progression of HCC is a result of a complex cellular system with reciprocal signaling. Three regulatory networks, PI3 K/Akt, ERK/MAPK, and JNK/c-Jun pathways, are confirmed to be dramatically induced by CXCL13 in prostate cancer [32]. Wnt/*β*-catenin signaling is also a very important pathway related to the tumor development. After Wnt binding to its receptor Frizzled (Fzd) and lipoprotein receptor-related protein 5/6 (LRP5/6), Axin/adenomatous polyposis coli (APC)/glycogen synthase kinase (GSK)3*β* complex is inactivated, which leads to the accumulation of *β*-catenin. Then *β*-catenin translocates to the nucleus and interacts with transcription factors of the T-cell factor (TCF) and lymphoid-enhancing factor (LEF) families, promoting the transcription of many oncogenic factors, such as c-MYC, cyclin D1, and VEGF [36, 37]. By preventing the Wnt signaling by its inhibitor Dkk-1 and stimulating this pathway by LiCl in HepG2 cells, we observed decreased levels of CXCL13 and its receptor CXCR5 in DKK-1 group, whereas increased expressions of these two factors was found in LiCl group. Likewise, Wnt/*β*-catenin transduction pathway was found to be stimulated by CXCL13 and blocked by dexamethasone as well. These data showed a positive relationship between CXCL13 and Wnt/*β*-catenin pathway in HCC and indicated that the mechanism of CXCL13 in promoting proinflammatory reaction might be mediated by the mutual feedback with Wnt/*β*-catenin signaling.

Considering CXCL13 plays a central role in the positioning, cooperation and activation of T and B cells within lymphoid and extralymphoid sites, especially in the differentiation of B cells into antibody-producing plasma cells [28], we also detected the effect of CXCL13 on interleukins secreted by T lymphocytes and IgG subclasses secreted by B lymphocytes. IL-12 is able to shift differentiation of CD4+ Th0 cells towards the Th1 phenotype [38], while IL-4 and IL-6 generally mediate the development of Th2 mediated diseases and regulate the Th1 responses by inhibition of interferon-*γ* (IFN-*γ*) production [39, 40]. IL-17 is a proinflammatory cytokine and required in vivo for the stabilization and proliferation of Th17 cells. It plays a very important role in chronic inflammatory state and stimulates the production of multiple chemokines, including CXCL1, CXCL8, CCL7, and CCL20 [41]. In PBMCs cultured with HepG2 supernatant, the expressions of IL-12 and IL-17 obviously showed an upregulation in CXCL13 group and a decreasing trend in dexamethasone group (*P* < 0.05), while minimal changes of IL-4 and IL-6 level were found in our experiment. The abnormally high production of IL-12 and IL-17, which presented Th1 and Th17 cell, respectively, indicated an immunological imbalance in CXCL13 stimulated group and that might be related to the HCC progression.

Compared with that in T lymphocytes, CXCL13 may have a crucial function in B lymphocytes as well. To clarify the effect of CXCL13 on B lymphocytes, we analyzed the proportions of IgG subclasses, which were reported to be associated with many cancers' progression [42, 43]. After stimulating B cells in vitro, we detected a higher IgG4/IgG total ratios in CXCL13 treated group compared with that in dexamethasone group or control. The ratios of IgG1, IgG2, and IgG3 showed no statistical significances in all groups. Our results suggested that CXCL13 might induce the imbalance of different subclasses secreted by B cells and mediated immunomodulatory activities in tumor microenvironment.

In conclusion, this study underlines the prognostic relevance of CXCL13 to HCC. We observed a considerable overexpression of CXCL13 in both liver cancer tissues and serum, and its level was positively correlated with serum ALT and AST. Furthermore, CXCL13 and Wnt/*β*-catenin signaling shared a positive feedback loop. Subsequently, we also found CXCL13 might function through activating Th1 and Th17 reaction and inducing IgG4 production. Thus CXCL13 may act as a promising prognostic marker and a potential therapeutic target in HCC.

## Figures and Tables

**Table 1:** The clinical characteristics of subjects.

Parameters	Group A Stages I-II *n* = 10	Group B Stage III *n* = 8	Group C Stage IV *n* = 14	HC *n* = 12
Age (years)	52 (46–67)	48 (44–68)	54 (50–75)	49 (43–74)

Gender: female/male	7/3	6/2	12/2	11/1

AFP (ng/mL)	58.2^*^ (21.7–83.3)	152.7^*^ (10.4–325.4)	194.6^*^ (6.3–810)	7.1 (1.3–11.1)

ALT (U/L)	67.3^*^ (18–243)	152.7^*^ (98.1–305.8)	215.2^*^ (145–712.6)	24.2 (13.6–27.8)

AST (U/L)	93.1^*^ (34–737)	183.4^*^ (79.2–385.2)	371^*^ (127.1–963.7)	22.4 (15.3–32.1)

Rate of high level CXCL13 (%)	20	37.5	64.3	—

Data shown are real case number or median (range).

Normal values: AFP: <25 ng/mL; ALT: <40 IU/L; AST: <40 IU/L.

HC: healthy control.

—: not available.

^*∗*^ *P* < 0.05 versus the HC.
